# Ambulatory Assessment of the Dynamic Margin of Stability Using an Inertial Sensor Network

**DOI:** 10.3390/s19194117

**Published:** 2019-09-23

**Authors:** Michelangelo Guaitolini, Federica Aprigliano, Andrea Mannini, Silvestro Micera, Vito Monaco, Angelo Maria Sabatini

**Affiliations:** 1 The BioRobotics Institute, Scuola Superiore Sant’Anna, 56127 Pisa, Italy; federica.aprigliano@santannapisa.it (F.A.); andrea.mannini@santannapisa.it (A.M.); silvestro.micera@santannapisa.it (S.M.); vito.monaco@santannapisa.it (V.M.); angelo.sabatini@santannapisa.it (A.M.S.); 2 Department of Excellence in Robotics & AI, Scuola Superiore Sant’Anna, 56127 Pisa, Italy; 3 Bertarelli Foundation Chair in Translational Neuroengineering, Center for Neuroprosthetics and Institute of Bioengineering, School of Engineering, Ecole Polytechnique Federale de Lausanne, 1015 Lausanne, Switzerland; 4 IRCCS Fondazione Don Carlo Gnocchi, 20148 Milan, Italy

**Keywords:** margin of stability, inertial sensors, gait analysis, stability

## Abstract

Loss of stability is a precursor to falling and therefore represents a leading cause of injury, especially in fragile people. Thus, dynamic stability during activities of daily living (ADLs) needs to be considered to assess balance control and fall risk. The dynamic margin of stability (MOS) is often used as an indicator of how the body center of mass is located and moves relative to the base of support. In this work, we propose a magneto-inertial measurement unit (MIMU)-based method to assess the MOS of a gait. Six young healthy subjects were asked to walk on a treadmill at different velocities while wearing MIMUs on their lower limbs and pelvis. We then assessed the MOS by computing the lower body displacement with respect to the leading inverse kinematics approach. The results were compared with those obtained using a camera-based system in terms of root mean square deviation (RMSD) and correlation coefficient (ρ). We obtained a RMSD of ≤1.80 cm and ρ ≥ 0.85 for each walking velocity. The findings revealed that our method is comparable to camera-based systems in terms of accuracy, suggesting that it may represent a strategy to assess stability during ADLs in unstructured environments.

## 1. Introduction

Falling is widely acknowledged as one of the major sources of morbidity and mortality, especially in elderly and fragile people [1]. It encompasses social diseases (e.g., lack of autonomy and independence, sedentary lifestyle, and psychological disorders) and represents a huge burden for healthcare systems [2,3]. The increasing life expectancy of the worldwide population is likely to further exacerbate the problems of falling. As such, in recent decades, several methods have been proposed to quantify the control individuals have over their balance during activities of daily living (ADLs) in order to screen those with a higher risk of falling [4].

Some authors focused their attention on the dynamic control of the whole-body center of mass (BCOM) relative to the base of support (BOS) during several ADLs (e.g., walking or counteracting external perturbations). Specifically, the condition for maintaining balance during an upright stance is that the vertical projection of the BCOM must remain within the boundaries of the BOS [5]. Pai and Patton highlighted that both the position and velocity of the BCOM with respect to the BOS are key aspects for balance control during dynamic motor tasks, such as walking [6]. To account for this issue, Hof and colleagues developed the dynamic margin of stability (MOS), defined as the distance between the extrapolated BCOM position (henceforth, the XCOM, which is the BCOM vertical projection adjusted by a factor depending on its velocity) and the boundaries of the BOS [7] (see Section 2.5 for further details). The MOS allows for a quantitative analysis of the dynamic control of the BCOM during each single walking step under the assumption of the pendulum-like oscillations of the musculoskeletal system [8]. Accordingly, this metric has previously been applied to the assessment of balance during walking control over rough surfaces [9], during direction changes [10], and while managing external perturbations [11,12,13].

One of the main limits of this approach is that the XCOM is estimated from data collected in structured experimental setups, usually provided with camera-based systems [8,10,11,12,13,14]. This methodological issue does not allow for investigating human stability outdoors or during ADLs, thereby leading scientists to request tasks that are carried out in unnatural conditions [15].

To overcome this limit, some research groups have recently estimated the dynamic MOS using only wearable sensors, that is, from inertial sensors and force-sensing resistors embedded in soles [16,17,18]. However, these approaches show limitations. Specifically, instrumented shoes can be bulky and can alter gait patterns [16,17]. Furthermore, instrumented shoes return 1D forces and moments that require subject-specific regression models to estimate 3D forces and moments, which are needed to estimate the MOS [16]. Finally, in the cited studies, inertial measurement units (IMUs) were mainly used to assess the BCOM velocity, but its displacement with respect to the leading foot was not accounted for.

Other groups used anthropometric-based approximations [17] to estimate the 3D displacement of the BCOM, which is necessary to assess the MOS. Arvin and colleagues [18] solved this issue by estimating the 3D displacement of the BCOM using an inertial unit placed on the pelvis. However, they did not evaluate the BOS from sensors; instead they used fixed values obtained by making the subject walk following tracks with both feet. In addition, although previous authors investigated the effects of various BOS widths on dynamic stability, the results only referred to the MOS in the medio-lateral direction. In addition, Schepers and colleagues proposed a method to estimate the BCOM during walking by combining information from shoes provided with force/moment sensors and IMUs [19]; furthermore, Guo and colleagues assessed the accuracy of the BOS estimation by combining signals from a network of IMUs during different body postures/motion [20]. Although relevant, in both of these studies, the authors did not estimate the MOS.

Finally, previous studies describing wearable sensor-based solutions for dynamic MOS estimation did not validate their results by means of a golden standard measurement tool (e.g., a stereo-photogrammetric system). In contrast, they compared the outcome of different wearable technologies in order to reduce the setup encumbrance [16], or investigate the effects of age, balance control [17,18], and walking conditions [18], without reporting information concerning the accuracy of the proposed method.

A short perspective of previous works regarding dynamic MOS evaluation through wearable sensors is reported in Table 1.

Nowadays, wearable sensors, such as magneto-inertial measurement units (MIMUs), represent a viable alternative to reliably track the kinematics of an individual’s body segments in daily life settings [21,22,23]. Compared to optical systems, wearable motion capture approaches feature simple equipment and low costs, although they are less accurate in reconstructing the motion kinematics and require specific sensor-fusion algorithms to overcome the shortcomings of each single sensing source within the MIMU hardware [24,25]. In this respect, several authors have demonstrated that specific kinematic patterns can be computed by integrating signals coming from MIMUs. For instance, errors of ≤3° and correlation coefficients of ≥0.8 were obtained while comparing joint angles assessed using both MIMU-based methods and optoelectronic systems [26,27,28]. Other researchers performed a trajectory reconstruction of feet and the BCOM using a small set of inertial units [19,29,30,31,32,33,34], achieving displacement errors of ≈3 cm between the MIMUs and camera-based systems. Finally, a recent study considering the reconstruction of the whole lower body kinematics of a gait reported a correlation coefficient greater than 0.9 when the outcome of the MIMU-based estimation was compared to that of a camera-based system.

These promising results allowed us to hypothesize that the dynamic stability, as assessed by the MOS, can be monitored by only using wearable sensors. Specifically, we hypothesized that a suitable network of MIMUs allows for accurate estimation of the MOS. To test this hypothesis, we developed a MIMU-based approach to estimate the MOS during walking through an inverse kinematics strategy and assessed the accuracy of the proposed approach by comparing the estimated MOS with that computed using data collected from a camera-based system. If accurate enough, the proposed approach is expected to both help clinicians and caregivers in rehabilitation procedures and patient monitoring, allowing for the assessment of balance and motion parameters in other fields of application where camera-based systems cannot be used easily (e.g., sports).

## 2. Materials and Methods

### 2.1. Participants, Experimental Setup, and Protocol

Six young (25 ± 2 years, two males) healthy subjects with comparable body masses (58.3 ± 7.4 kg) and heights (1.67 ± 0.10 m) volunteered to participate in this study. The subjects had no known history of postural, musculoskeletal, or neurological diseases.

**Figure 1:**
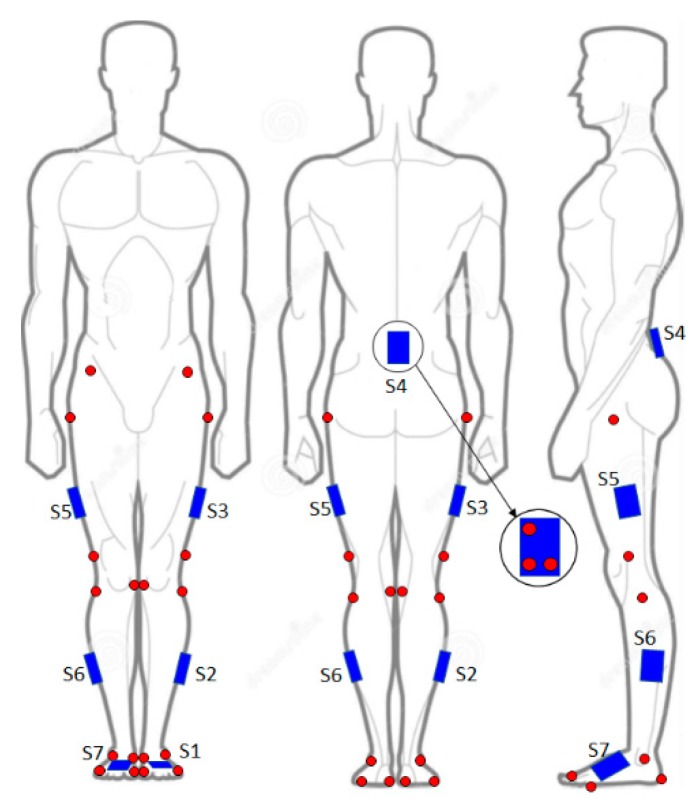
Markers and inertial units’ placement. Each inertial unit was equipped with three markers (red dots).

Participants were asked to walk on a treadmill while donning 41 reflective markers and seven MIMUs (Pivot, TuringSense Inc., Santa Clara, California) placed on lower limbs (pelvis, thighs, shanks, and feet), as shown in Figure 1. Each Pivot unit was embedded with an Invensense MPU9250 unit, which contained a triaxial gyroscope (± 2000 deg/s) and a triaxial accelerometer (± 16 g, where g = 9.81 m/s^2^) [35], as well as a triaxial magnetometer Asahi AK8963 (± 4800 µT) [36]. The output of this sensor set was used to feed the on-board implementation of the Kalman filter-based orientation estimator. 

Twenty-two markers were located on the following body landmarks: the right and left anterior superior iliac spines, greater trochanters, lateral and medial epicondyles of the femurs, heads of fibulas, lateral and medial malleoli, heels, and first and fifth metatarsal heads (Figure 1). In addition, each MIMU was mounted on a 3D-printed plastic support, which also carried three unaligned reflective markers (Figure 1).

The marker set was a revised version of the Plug-in Gait protocol. Specifically, starting from the Plug-in Gait, we added markers on the medial epicondyles of both femurs and medial malleoli. In addition, both thighs and shank bars, and markers on the posterior iliac spines, were replaced by markers on the 3D-printed plastic supports embedded with MIMUs, as in Figure 1.

The 3D trajectory of all markers was captured by a six-camera-based system (Vicon, Oxford, U.K.) with a sample rate of 100 Hz. MIMU data were also recorded at 100 Hz. The estimated maximum measurement was ≤1 mm.

Before starting the experimental sessions, data were collected while the subjects kept their orthostatic upright stance for ≈10 s for calibration purposes (see Section 2.7). The whole experimental protocol then involved six walking sessions, randomly ordered, at three speeds (2.5, 3.5, and 4.5 km/h) with two repetitions each. For each session, the participants were asked to start walking after performing a couple of jumps from a steady stance for off-line synchronization of data streams recorded by cameras and MIMUs (see Section 2.3). MIMUs were also calibrated using the standard in-field procedures implemented in the Pivot units, as specifically provided by the manufacturer.

The participants were then allowed to walk for about three minutes to familiarize themselves with treadmill walking [37]. After that, the participants continued walking for two additional minutes and the data recorded during this time window were retained for analysis. Between two consecutive sessions, subjects could rest on a chair for as long as they required to be ready for the next session.

All research procedures were in accordance with the Declaration of Helsinki [38] and were approved by the Institutional Ethics Committee of the IRCCS (Istituto di Ricovero e Cura a Carattere Scientifico) Fondazione Don Carlo Gnocchi.

### 2.2. Data Pre-Processing

Data recorded by cameras were low-pass filtered using a second order, zero-lag, Butterworth filter with a cut-off frequency at 6 Hz [39]. The 3D components of both linear acceleration and angular velocity, the magnetic field, and the resulting orientation of each MIMU were automatically collected by the Pivot software. Noticeably, the orientation of each sensor was computed in accordance with the method described in [40] and reported using quaternion notation.

### 2.3. Synchronization and Time Events

Datasets from the camera system and MIMUs were time-aligned by assessing the time-lag between them. Specifically, we computed the cross-correlation of the second time derivative of the vertical component of one marker on the MIMU on the right foot with the vertical component of the acceleration detected by the same MIMU within a 5-s time window, including the two initial jumps. The time-lag between data streams coincided with the abscissa of the maximum of their related cross-correlation function.

The data recorded by MIMUs on the participant’s feet were also used to detect heel strike (HS) events. A HS was identified as the negative peak of the angular velocity along the mediolateral axis, which preceded the signal plateau occurring at the flat-foot event, in accordance with previous literature [41].

### 2.4. Reference Frames

**Figure 2:**
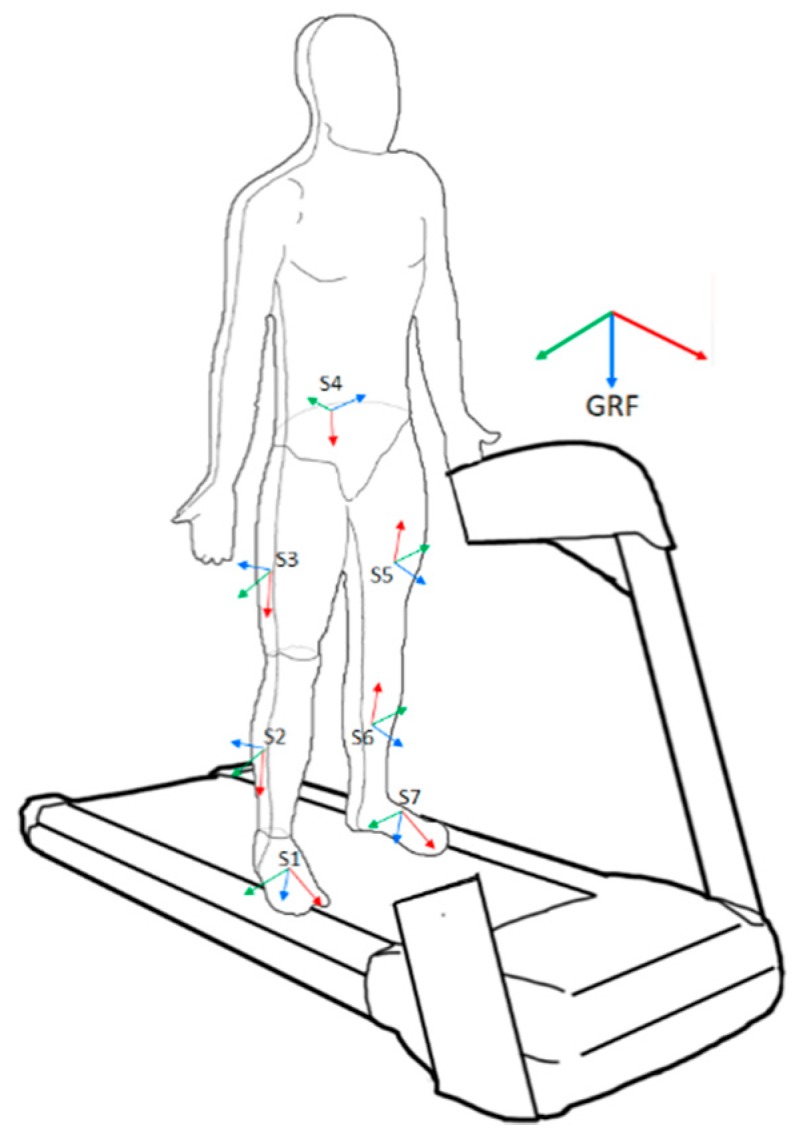
MIMUs’ local reference frames and the global reference frame (GRF). The red, green, and blue axes represent the *x*-, *y*-, and *z*-axes, respectively.

The data recorded during the experimental sessions referred to two different reference frames (Figure 2):

1. The global reference frame (GRF), where the *x*-axis was aligned with the longitudinal axes of the treadmill, forward oriented; the *z*-axis was vertical, downward oriented; and the *y*-axis resulted from the right-hand rule as a vector directed toward the right side of the treadmill. This was the camera-based reference frame and the 3D trajectories of all markers referred to this reference frame.
2. A local reference frame for each body segment, set according to the axes of the related MIMU. Noticeably, the output of each MIMU was the quaternion-based orientation expressed from its navigation reference frame to the local reference frame (i.e., the global reference frame of the MIMU used axes aligned with magnetic North, gravity, and the axis orthogonal to these two in accordance with the right-hand rule); it should be noted that the navigation reference frame was defined during the MIMU calibration procedure, and due to non-idealities, each MIMU produced a different navigation reference frame

Each MIMU was equipped with three markers, allowing us to estimate its orientation with respect to the GRF (Figure 1). Hence, for each MIMU, we could estimate the relationship between the local (i.e., MIMU-based) reference frames and the GRF (outcome of the camera-based system). 

It should be noted that kinematic chain reconstruction through inertial sensors relies on the assumption that the sensor orientations refer to the same navigation frame. This fact reflects an ideal condition that is nearly impossible to replicate. Therefore, it is necessary to perform a calibration. One of the possible methods to achieve this relies on the known GRF provided by an external measurement system, such as our camera-based system. For this reason, we projected the output of the MIMUs into the GRF. This procedure is equivalent to a sensor-to-segment (STS) calibration performed with external references, as in Teufl et al. [42]. Accordingly, we could handle camera- and MIMU-based data in the same reference frame.

STS can also be performed without the help of an external reference, extracting the required information using specific motions and poses [43,44]. Hence, functional STS procedures could easily be implemented in our method, allowing us to obtain a lower body kinematic reconstruction without any reference from cameras. However, this is currently beyond the aim of the presented method, namely, finding a way to compute MOS using a MIMU network and validating the method against a gold reference standard provided by camera-based data.

### 2.5. Margin of Stability

The aim of the study consisted of assessing and comparing the MOS along *x*- and *y*-axes (AP and ML axes, respectively), using both camera-based and MIMU data. Briefly, the MOS represents the relative distance between the XCOM and the boundaries of the base of support [7]. In this study, we refer to the MOS as assessed in the sagittal (AP) and frontal (ML) plane, computed as follows:

(1) $$\mathrm{MOS}=U_{\mathrm{MAX}}-\mathrm{XCOM}$$

where U_MAX_ is the boundary of the BOS. Concerning the AP direction, the U_MAX_ coincided with the first metatarsal head of the leading foot; for the ML direction, it coincided with the fifth metatarsal head of the leading foot.

The XCOM is the BCOM adjusted by its velocity along the AP and ML axes and can be estimated as follows:

(2) $$\mathrm{XCOM}\;=\;\mathrm{BCOM}\;+\;\dot{\mathrm{BCOM}}/\sqrt{\frac{g}{h_{\mathrm{BCOM}\;}}\;}$$

where BCOM and $\dot{\mathrm{BCOM}}$ are the position and velocity of the BCOM, respectively,$\;h_{\mathrm{BCOM}}$ is the distance between the BCOM and the floor while subjects maintained an unperturbed upright stance, and *g* is the gravitational acceleration (9.81 m/s^2^).

In this study, we assumed that the BCOM coincided with the midpoint between the two bottom markers placed on the support for the pelvis MIMU. In addition, we computed the MOS behavior in a time window starting with the HS of the leading limb and ending with the HS of the contralateral one (i.e., one step).

### 2.6. MOS Estimation Using Camera-Based Data

To compute the MOS using camera-based data, we retained the markers located on the leading foot and the 3D trajectory of the midpoint between the two bottom markers placed on the support for the pelvis MIMU. This point was entitled SACRUM. Specifically:

- The BOS boundaries along the AP direction coincided with the *x* component of the first metatarsal head of the leading foot, while the BOS boundary along the ML direction coincided with the *y* component of the fifth metatarsal head of the leading foot.
- The BCOM position along both axes (i.e., AP and ML) coincided with the *x* and *y* components of the marker on the SACRUM, respectively.
- The $\dot{\mathrm{BCOM}}$ was computed as the first time derivative (backward difference method) of the BCOM trajectory in both frontal and sagittal planes that were smoothed using a moving average filter across a time window of 20 samples to remove high-frequency noise.

### 2.7. MOS Estimation Using MIMU Data

To estimate the MOS using MIMU data, we computed the relative distance between the BOS boundaries (i.e., first and fifth metatarsal heads) and the XCOM by combining subject-specific anthropometrical measurements and the orientation of each body segment. The former data were collected during a static trial, while the latter ones were collected during walking tasks using MIMUs.

In detail, we defined a kinematic model that described the leading limb as a mechanical chain accounting for four body segments: foot, shank, thigh, and pelvis. We assumed that leg joints were spherical and were located as follows:

- The ankle joint coincided with the midpoint between the medial and lateral malleolus.
- The knee joint coincided with the midpoint between the medial and lateral epicondyles of the femur.
- The hip joint coincided with the position of the acetabular cup, assessed as described elsewhere [45].

**Figure 3:**
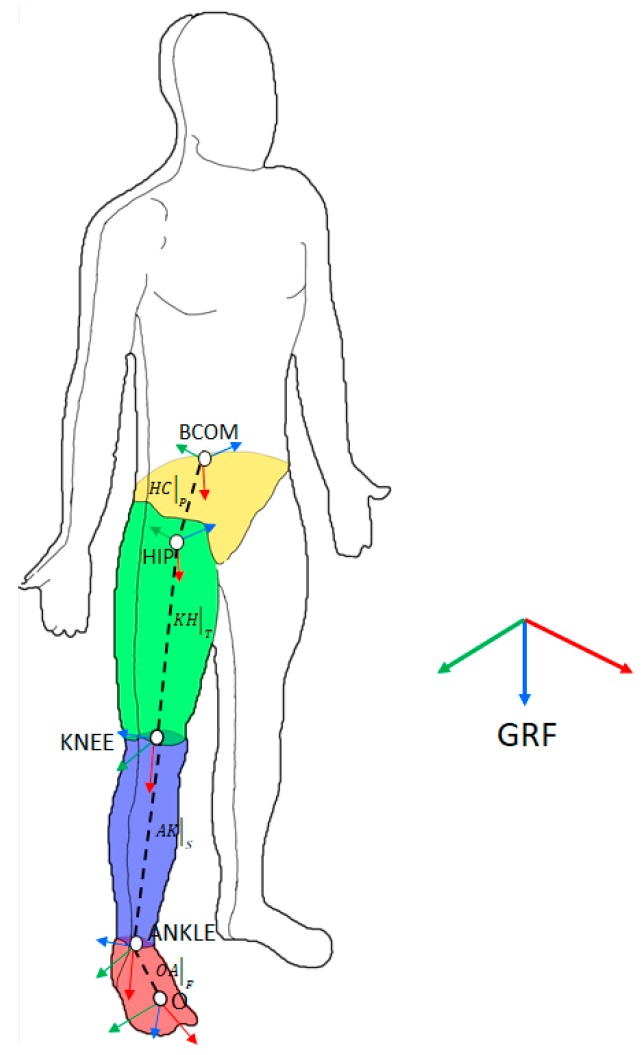
Inverse kinematic reconstruction scheme. BCOM—whole-body center of mass; H—hip; K—knee; A—ankle; O—origin.

For each body segment, we set a reference frame whose orientation with respect to the GRF was defined using MIMU’s outcome, while the origins, from the shanks to the pelvis, coincided with the joints (respectively, *A,* ankle; *K*, knee; *H*, hip), as reported in Figure 3. As far as the foot was concerned, the origin of its local reference frame, namely *O*, was one of the three markers on the MIMU.

The COM trajectory with respect to the foot origin could hence be computed as follows (Figure 3):

(3) $$\bar{\mathrm{BCOM}}{|}_{\mathrm{GRF}}=R_{F\to \mathrm{GRF}}\cdot \bar{\mathrm{OA}}{|}_{F}+R_{S\to \mathrm{GRF}}\cdot \bar{\mathrm{AK}}{|}_{S}+R_{T\to \mathrm{GRF}}\cdot \bar{\mathrm{KH}}{|}_{T}+R_{P\to \mathrm{GRF}}\cdot \bar{\mathrm{HB}}{|}_{P}$$

where $R_{F\to \mathrm{GRF}}$, $R_{S\to \mathrm{GRF}}$, $R_{T\to \mathrm{GRF}}$, and $R_{P\to \mathrm{GRF}}$ are the rotation matrices from the local frames (i.e., F, foot; S, shank; T, thigh; P, pelvis) to the GRF and ${\bar{\mathrm{OA}}|}_{F}$, ${\bar{\mathrm{AK}}|}_{S}$, ${\bar{\mathrm{KH}}|}_{T}$, and ${\bar{\mathrm{HB}}|}_{P}$ are vectors connecting the origins of reference frames referring to two consecutive body segments (B stands for BCOM and represents the point corresponding to the BCOM). Each of the rotation matrices reported in Equation (3) were obtained from the quaternion output of the related MIMU (expressed as $q=q_{1}x+q_{2}y+q_{3}z+q_{4}$) as follows:

(4) $$R=[\begin{array}{ccc} q_{4}^{2}+q_{1}^{2}-q_{2}^{2}-q_{3}^{2} & 2(q_{1}q_{2}+q_{4}q_{3}) & 2(q_{1}q_{3}-q_{4}q_{2})\\ 2(q_{1}q_{2}-q_{4}q_{3}) & q_{4}^{2}-q_{1}^{2}+q_{2}^{2}-q_{3}^{2} & 2(q_{2}q_{3}+q_{4}q_{1})\\ 2(q_{1}q_{3}+q_{4}q_{2}) & 2(q_{2}q_{3}-q_{4}q_{1}) & q_{4}^{2}-q_{1}^{2}-q_{2}^{2}+q_{3}^{2} \end{array}]$$

The coordinates of ${\bar{OA}|}_{F}$, ${\bar{AK}|}_{S}$, ${\bar{KH}|}_{T}$, and ${\bar{HB}|}_{P}$ in their related local reference frame were computed off-line from data collected by the camera-based system while subjects maintained the unperturbed orthostatic posture. Furthermore, we also computed the coordinates of the first and fifth metatarsal heads with respect to the foot reference frame to identify BOS boundaries along the AP and ML directions.

Finally, we used the *x* and *y* components of $\bar{\mathrm{BCOM}}{|}_{\mathrm{GRF}}$ and their first derivative to estimate the XCOM components. We then estimated the MOS as the distance between the XCOM and BOS boundaries along the AP and ML directions.

### 2.8. Outcome Measures and Statistical Analysis

The 3D trajectory of the ankle, knee, hip, and BCOM, as well as the MOS along the AP and ML directions, were computed from data provided by MIMUs and compared to those obtained from the camera-based system by using the root mean square deviation (RMSD) and the Pearson’s coefficient (ρ) as performance metrics. The approach based on camera data was considered the gold reference, thus the RMSD and ρ allowed for assessment of the discrepancy existing between approaches.

Data normality was tested and confirmed using the Shapiro–Wilk test. Then, a repeated-measures one-way analysis of variance (ANOVA) was performed to investigate the effect of different walking speeds (namely 2.5, 3.5, and 4.5 km/h) on the MOS estimation accuracy. The variable of the test was the MOS RMSD; the null hypothesis was that the walking speed does not affect MOS estimation accuracy. Statistical significance was set at *p* < 0.05. 

We had two trials available for each subject, for each walking speed, for a total number of twelve observations for each walking speed. The Bonferroni post-hoc test was performed after the ANOVA.

Statistical analysis was performed using MATLAB (2017b The MathWorks, Inc., Natick, MA, USA).

## 3. Results

### 3.1. Kinematic Chain Reconstruction

**Figure 4:**
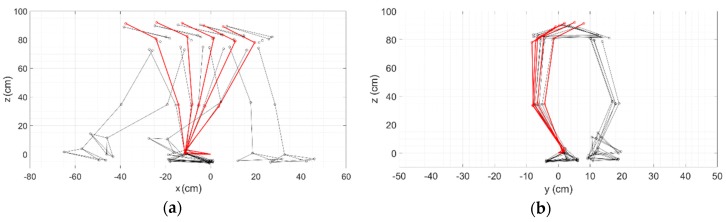
Lower body reconstruction through the camera-based system (black structure) with an overlapping leg reconstruction from the MIMUs (red structure): (**a**) lateral view, and (**b**) frontal view.

Figure 4 shows a representative example of the 3D trajectory of the leading limb during one step, estimated using both datasets (i.e., camera- and MIMU-based), and centered in *O*.

Kinematic patterns were estimated using both motion capture systems and were related to observed body landmarks (i.e., ankle, knee, hip, and BCOM) correlations (ρ ≥ 0.92, always).

**Figure 5:**
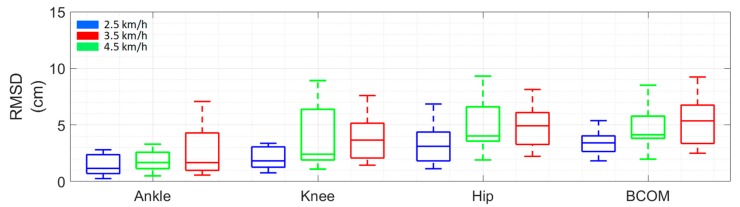
Root mean square deviation (RMSD) between trajectories computed using MIMU- and camera-based data for each walking velocity.

The results, in terms of median RMSD, were always lower than 5.11 cm (Figure 5), despite the reconstruction suffering an accuracy-decreasing effect from the distal to the proximal landmarks of the kinematic chain. The RMSD values along the kinematic chain were in accordance with previous findings [21].

### 3.2. Margin of Stability

**Figure 6:**
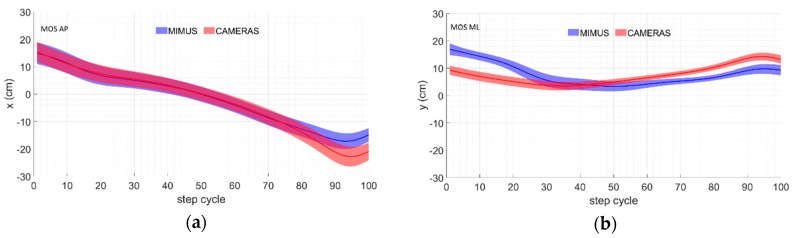
MOS trends computed using MIMU- and camera-based data along the (**a**)anteroposterior (AP) and (**b**) mediolateral (ML) directions.

Figure 6 shows the time course (average and standard deviation) of the MOS, BOS, XCOM, and BCOM computed using both datasets (i.e., camera-based to the right and MIMU-based to the left) along a time-normalized single step.

**Figure 7:**
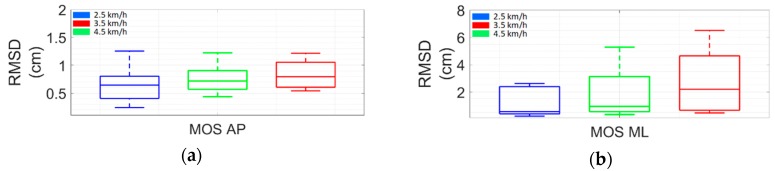
RMSD between MOS computed using MIMU- and camera-based data for each walking velocity along the (**a**) AP and (**b**) ML directions. Take note that the scale of the vertical axis scale is different between panels.

The RMSD obtained for the MOS estimation (Figure 7) was lower than that obtained for the trajectory estimation (Figure 5) and ρ was always higher than 0.85. It should be noted that the errors in the MOS estimation were concentrated mainly in the ML direction. This fact may be related to the segment orientation estimation, which was less accurate in terms of what it regarded as the rotation around the anteroposterior axis (Figure 6). Nevertheless, the overall displacement estimation result was always 1.80 cm.

Statistical analysis showed that the adopted walking velocities did not significantly affect the MOS estimation accuracy.

## 4. Discussion

This study aimed to assess the accuracy of a MIMU-based approach while estimating both the position of the ankle, knee, and hip joints, and the dynamic MOS during human walking. The proposed method relies on inverse kinematics to estimate the position and orientation of lower limb segments with respect to the leading foot in order to compute the MOS.

The results revealed that the accuracy of the proposed method while estimating lower limb landmarks is comparable with previous literature in terms of correlation coefficients and RMSD [21,23] (more details in Section 4.1). For the MOS estimation, the proposed method produced a median RMSD of 0.74 cm (75th percentile = 1.80 cm; 25th percentile = 0.49 cm) and a correlation coefficient always higher than 0.85 when compared with data provided by a camera-based system. Considering the AP and ML directions, we obtained median RMSDs of 0.68 cm (75th percentile = 0.89 cm; 25th percentile = 0.57 cm) and 0.94 cm (75th percentile = 2.89 cm; 25th percentile = 0.47 cm), respectively.

### 4.1. Accuracy of Joint Position Estimation

The results revealed that the accuracy of the proposed method when estimating lower limb landmarks is comparable with previous literature in terms of the RMSD and correlation coefficients [21,23]. More precisely, we achieved median RMSDs of 1.26 cm (75th percentile = 2.02 cm; 25th percentile = 0.88 cm) for the ankle, 2.14 cm (75th percentile = 3.56 cm; 25th percentile = 1.53 cm) for the knee, 3.85 cm (75th percentile = 4.58 cm; 25th percentile = 2.47 cm) for the hip, and 3.82 cm (75th percentile = 5.11 cm; 25th percentile = 2.80 cm) for the BCOM.

The accuracy was in line with that obtained by Fasel and colleagues [21], since they reported a mean error of 2.80 ± 0.50 cm on the most proximal point while we obtained a median error of 1.26 cm. They also reported a mean error of 10.90 ± 2.90 cm on the most distal point, while we obtained a median error of 3.82 cm.

In terms of correlation coefficients, we obtained a correlation coefficient that was always higher than 0.92, reflecting a good match between reconstructed trajectories and the reference. Our results are comparable to results obtained by Ahmadi and colleagues, who reported correlation coefficients that were always higher than 0.94 for each considered body landmark [23]. 

### 4.2. Accuracy of Estimation of MOS

The second main objective of this study consisted of assessing the accuracy of the MOS estimation using multiple MIMUs when compared to that computed by using data from a camera-based system. To the best of our knowledge, this was the first study that compared two methods (i.e., MIMUs-based versus camera-based) to assess the MOS.

Our results, in terms of deviations between approximate and reference MOS, were lower than typical MOS variability computed using cameras [8,9]. In fact, previous authors reported that MOS variability during steady walking ranges between 1.5 and 3 cm along the ML direction and 1.5 and 2 cm along the AP direction [9]. The adopted method was not affected by accuracy loss due to faster walking speeds.

Thus, we can conclude that an overall error lower than 2 cm may be considered acceptable for the stability assessment.

### 4.3. Limits

The proposed method is affected by a few methodological limitations. Our approach is currently only reliable in structured environments with the support of a camera-based system, which is due to two reasons. First, the requirement of knowing anthropometrical measures from camera-based data acquired during a static trial without kinematic chain reconstruction would not be possible. Moreover, cameras are needed for sensor-to-segment calibration. As already mentioned (Section 2.4), STS calibration procedures can be performed through functional movements without camera data; these procedures will be included in future developments. 

In addition, it should be considered that many MIMUs are usually required to properly assess body displacement, and consequently, to assess the dynamic MOS. For the lower body kinematic chain, we employed seven MIMUs and this network may be bulky for daily use. However, the number of MIMUs may be reduced. This hypothesis relies on the evidence that the kinematics of lower limb segments is constrained by the anatomical joints. In addition, several studies have demonstrated that the kinematics of lower limb segments is significantly correlated during several voluntary and involuntary walking-related motor tasks [46,47,48,49]. Accordingly, a smaller network of MIMUs is expected to allow for an accurate estimation of both limb joints and MOS. Further investigations that go beyond the aims of this preliminary study are needed to test this hypothesis.

Finally, another limit of this study is related to the small number of enrolled subjects, resulting in the limited power of the statistical findings. However, we would like to remark that this paper was intended to be a preliminary study to verify whether the proposed approach was actually feasible. Therefore, the number of enrolled subjects was selected in accordance with a previous similar proof-of-concept study [16]. Further investigations involving more subjects are required to strengthen the outcomes of our preliminary analysis.

## 5. Conclusions

We have presented a computationally simple method to assess the dynamic MOS of a gait using MIMUs. Our approach only relies on body-worn inertial sensors placed on the pelvis and both left and right thighs, shanks, and feet, and can assess BCOM position with respect to the leading foot (left and right foot, alternately) by using an inverse kinematics approach. The lower body kinematic chain was reconstructed by means of orientation information provided using MIMUs and anthropometric information provided by a static trial with the camera-based system. 

The results showed that the proposed method is reasonably accurate in estimating lower limb landmarks. In addition the proposed method allows for estimating the MOS with a good accuracy compared to that obtained by a camera-based system, both in terms of RMSD (AP direction: median RMSD = 0.68 cm, 25th percentile = 0.57, 75th percentile = 9.8; ML direction: median RMSD = 0.94 cm, 25th percentile = 0.47, 75th percentile = 2.89) and correlation coefficient (always ≥0.85).

This study was intended to present preliminary results and future research will focus on the limitations that affect the proposed approach; namely, the unchecked sensor placement and the need to know anthropometric information from one static camera-based trial. Reducing the number of sensors in our network would also significantly improve its eligibility for any kind of application.

We believe that the assessment of stability through wearable sensors without altering subject movements may be extremely significant in many application fields such as assistance, prosthetic control, and injury prevention in sport activities. Possible research scenarios in the near future may relate to the application of this approach in different contexts/environments, such outdoor gait measurement or in the presence of external perturbation.

## Figures and Tables

**Table 1:** Summary of works that performed MOS assessment using wearable systems and a comparison of their methods with the method proposed in this study.

Methods	Setup	MOS Directions	Camera Validation	Aim
Refai et al. [16]	Instrumented shoes; IMU on each forefoot; ultrasound system	Frontal	No	Compare two wearable methods; validate the less bulky
Van Meulen et al. [17]	Instrumented shoes; ultrasound system	Frontal	No	Investigate MOS correlation with clinical stability scale
Arvin et al. [18]	Instrumented shoes; IMU on pelvis	Sagittal	No	Investigate effect of BOS width on MOS
Presented method	Seven MIMUs on feet, shanks, thighs, and pelvis	Frontal and Sagittal	Yes	Reconstruct MOS using MIMUs and validate the method using a camera-based system
